# Implementing evolving Fabry disease management recommendations in resource-constrained settings: lessons from South Africa

**DOI:** 10.1186/s13023-026-04542-9

**Published:** 2026-08-12

**Authors:** Helen Louise Malherbe, Christian J. Hendriksz, Barend Christiaan Vorster

**Affiliations:** Centre for Human Metabolomics, Desmond Tutu School of Medicine, Faculty of Health Sciences, North-West University, 11 Hoffman Street, Potchefstroom, North West Province 2531 South Africa

**Keywords:** Fabry disease, Lysosomal storage disorder, Rare diseases, Enzyme replacement therapy, Early diagnosis, Risk stratification, Disease monitoring, South Africa, Real-world evidence, Resource-constrained settings

## Abstract

Fabry disease (FD) is a rare X-linked lysosomal storage disorder caused by deficient activity of the enzyme α-galactosidase A, resulting in glycosphingolipid accumulation and progressive involvement of multiple organ systems, particularly the kidneys, heart and cerebrovascular system. Clinical manifestations are highly heterogeneous, with disease onset ranging from early childhood to late adulthood depending on sex, genotype and phenotype. Although rare, FD can result in substantial morbidity and premature mortality when diagnosis is delayed or management is suboptimal. International registry data suggest that diagnostic delays of more than a decade are common, with many patients presenting with advanced organ involvement before treatment is initiated. Delayed diagnosis and late initiation of therapy may be associated with irreversible organ damage and substantial downstream healthcare use related to dialysis, cardiac complications and stroke. Earlier recognition may improve the opportunity for clinically beneficial intervention; however, whether earlier initiation of high-cost disease-specific therapy represents a cost-effective use of resources depends on phenotype, disease stage, expected treatment effect, treatment cost and the opportunity costs within the relevant healthcare system. Over the past decade, the international evidence base guiding FD management has evolved substantially. Contemporary consensus recommendations increasingly emphasise earlier initiation of disease-specific therapy, improved recognition of clinically significant disease in females, genotype-informed treatment strategies, and risk-stratified approaches to monitoring and longitudinal care. Advances in biomarkers, molecular diagnostics and cardiac imaging have further refined approaches to disease assessment and treatment decision-making. This commentary highlights key developments in the international management of FD since the publication of South African recommendations in 2015 and considers their implications for rare disease care in resource-constrained health systems. It proposes an adaptive, resource-stratified implementation framework that integrates evolving international recommendations with transparent treatment prioritisation, longitudinal monitoring, local real-world evidence generation and periodic reassessment. The framework recognises that treatment effects differ by phenotype, sex, organ involvement and disease stage, and that decisions concerning high-cost disease-specific therapies must consider expected benefit, uncertainty, equity, affordability and opportunity cost.

## Background

Fabry disease (FD) is a rare X-linked lysosomal storage disorder (LSD) characterised by progressive involvement of mainly the kidneys, heart and cerebrovascular system. Clinical manifestations are highly heterogeneous, with disease onset ranging from early childhood to late adulthood depending on sex and phenotype. Although rare, FD can result in substantial morbidity and premature mortality when diagnosis is delayed or management is suboptimal [1]. International registry data suggest that diagnostic delays of more than a decade are common, with many patients presenting with advanced organ involvement before treatment is initiated [2]. Delayed diagnosis and initiation of therapy after irreversible organ damage has developed may limit the potential for disease modification. Advanced renal, cardiac and cerebrovascular complications are also associated with substantial healthcare resource use and costs [3]. Earlier recognition may increase the opportunity for clinically beneficial intervention, but the economic value of high-cost disease-specific therapy cannot be inferred from the costs of advanced complications and requires context-specific economic and budget-impact assessment [4]. 

Like many rare diseases, FD is associated with substantial diagnostic delay, fragmented multidisciplinary care and inequitable access to disease-specific therapies, particularly in low- and middle-income countries (LMICs). These challenges are increasingly recognised within broader international rare disease policy discussions concerning access, sustainability and health system preparedness [5]. Within this context, FD provides a useful exemplar through which to examine broader implementation challenges associated with rare disease care in resource-constrained settings.

In South Africa (SA), recommendations for the diagnosis and management of FD were published in 2015, providing an important framework for clinical care within the constraints of the local healthcare system [6]. These recommendations outlined approaches to diagnosis, baseline assessment and longitudinal monitoring, and established organ-based criteria for initiating enzyme replacement therapy (ERT). This represented an important step toward standardising care for patients with FD in the country.

Over a decade later, the international landscape of FD management has evolved substantially. International patient registries and longitudinal natural history studies have improved understanding of disease progression, treatment response and disease heterogeneity. Advances in molecular diagnostics, biomarkers and cardiac imaging have contributed to more individualised approaches to treatment initiation, monitoring and risk stratification [2, 7–9]. At the same time, uncertainty persists regarding the magnitude of long-term treatment benefit across different phenotypes, sexes, organ systems and stages of disease [10, 11]. These developments raise important questions about how emerging evidence should inform clinical practice and the prioritisation of high-cost care in SA and other resource-constrained settings.

Although this commentary focuses on SA, the proposed resource-stratified approach may also be relevant to other LMICs managing persistent high-burden health needs alongside costly therapies for rare genetic conditions.

## How Fabry disease management has evolved

### Earlier treatment strategies

Advances in the management of FD are summarised in Table 1. Although earlier guidelines in SA [6] recognised that treatment is most effective when initiated early in the disease course, the biomarkers and imaging modalities available at the time, including proteinuria, glomerular filtration rate, electrocardiogram (ECG) and echocardiography, were not specific to FD and largely reflected established organ damage. As a result, treatment initiation for FD was historically guided by the presence of advanced renal or cardiac involvement.

While these markers remain important for assessing disease severity and monitoring treatment response, reliance on them may delay treatment consideration until tissue injury is established. More recent consensus recommendations support earlier consideration of disease-specific therapy in appropriately selected individuals, particularly those with classic pathogenic variants and early evidence of Fabry-attributable organ involvement [7–9]. Treatment decisions should nevertheless integrate genotype, phenotype, biomarkers, imaging findings, disease trajectory and individualised assessment of likely benefit rather than assuming uniform benefit from early treatment.

Evidence suggests that renal outcomes during ERT are less favourable in patients with substantial baseline proteinuria or impaired renal function, while cardiac responsiveness may be reduced once advanced myocardial fibrosis has developed [12–15]. Longitudinal real-world evidence also indicates that outcomes and treatment response vary according to sex, phenotype, baseline organ involvement and competing risk factors [10, 16]. These findings support timely diagnosis and risk-stratified treatment assessment, while cautioning against simple treatment rules based on genetic confirmation or age alone.

Contemporary guidance emphasises improved detection of early pathological change using disease-specific biomarkers, including globotriaosylsphingosine (lyso-Gb3), and cardiac magnetic resonance imaging (MRI) with late gadolinium enhancement and T1 and T2 mapping [2, 9]. These modalities can support individualised risk assessment, but access is limited in many LMIC settings and may differ substantially between public and private sectors in dual or mixed healthcare systems. Resource-stratified pathways should identify feasible baseline alternatives, specify referral indications for advanced investigations and avoid making access to a single specialised test an absolute prerequisite for clinically necessary care.

From a health-system perspective, the reduced potential for disease modification after advanced organ damage has developed should be considered when assessing the expected benefit and value of initiating or continuing high-cost disease-specific therapy. Earlier intervention may reduce downstream complications and associated healthcare costs; however, its overall economic value should be established through context-specific economic and budget-impact analyses [4]. 

### Recognition of disease in females

Fabry disease was historically regarded primarily as a disorder affecting hemizygous males, with heterozygous females often considered asymptomatic carriers. Longitudinal cohort and registry data demonstrate that many females develop clinically significant cardiomyopathy, renal disease or cerebrovascular complications, with substantial variability in expression and progression [2]. This heterogeneity is influenced by X-chromosome inactivation, genotype, age and other biological and clinical factors [17]. 

Females frequently lack the classical early manifestations of FD, such as acroparesthesia, hypohidrosis and angiokeratoma, and may present later in life, often at a stage when common multifactorial conditions associated with cardiac and renal disease become increasingly prevalent, with less consideration of FD in the differential diagnosis. Vigilance and the identification of affected females through cascade screening are therefore of particular importance. Once a genetic diagnosis has been established, meticulous baseline assessment and longitudinal monitoring using disease-specific biomarkers and imaging modalities are essential to enable early detection of organ involvement and guide timely initiation of disease-specific therapy.

### Genotype-informed care

Advances in molecular genetics have improved the understanding of genotype–phenotype relationships in FD. International recommendations increasingly distinguish between classic pathogenic variants, later-onset variants and variants of uncertain significance, enabling more individualised approaches to treatment initiation, longitudinal monitoring and family counselling [8]. These developments underscore the importance of careful genetic interpretation and multidisciplinary expertise in evaluating patients with suspected FD. For patients with amenable GLA variants, migalastat provides a genotype-specific alternative to ERT, illustrating how variant interpretation increasingly informs treatment selection [18]. 

**Table 1:** Key areas in which international Fabry disease management recommendations have evolved since the 2015 South African guidance

Domain	2015 South African guidance (Bhengu et al., 2015)	Developments in international recommendations
**Treatment initiation**	Organ-based initiation criteria, generally using clinical evidence of renal, cardiac or neurological involvement.	Earlier, individualised treatment assessment incorporating genotype, phenotype, early disease indicators, expected benefit and disease stage.
**Disease in females**	Recognition that heterozygous females may be clinically affected, with treatment based on evidence of organ involvement.	Greater recognition of clinically significant disease in females, with phenotype- and risk-informed treatment decisions.
**Genotype interpretation**	Limited stratification by mutation type.	Greater use of genotype–phenotype correlations to guide monitoring and treatment decisions.
**Monitoring strategies**	Structured multisystem monitoring schedules.	More personalised monitoring incorporating phenotype, genotype, disease severity, treatment status and biomarker burden.
**Biomarkers and imaging**	Limited emphasis on emerging biomarkers and advanced imaging.	Expanded role for cardiac MRI and biomarkers such as lyso-Gb3, while recognising access limitations.
**Care Model**	Specialist-led care with structured multisystem assessment.	Longitudinal multidisciplinary care integrated with rare disease data systems, treatment review and outcome evaluation.

### Risk-stratified monitoring

Given the marked phenotypic heterogeneity in FD, recent recommendations increasingly advocate personalised, risk-stratified monitoring. Follow-up intensity should be tailored to phenotype, genotype, disease severity, biomarker burden, organ involvement, treatment status and estimated risk of progression [2, 8, 9]. This may focus specialist resources on individuals at greatest risk while reducing low-value investigations in lower-risk individuals.

## Implementing evolving Fabry disease care in resource-constrained health systems

Investigation schedules and treatment thresholds developed in well-resourced settings may not be directly transferable to resource-constrained health systems. Implementation requires earlier recognition of clinically significant disease, prioritisation of investigations and treatment according to expected benefit, and explicit consideration of opportunity costs and competing health-system priorities.

These considerations align with broader South African priorities to strengthen rare-disease diagnostic capacity, referral pathways, multidisciplinary care and equitable access to specialised services and high-cost therapies [19]. Global attention to these issues has increased following ratification of the 2025 World Health Assembly resolution on rare diseases, which calls for coordinated national and international action to improve diagnosis, care, treatment, data systems and inclusion [5]. 

## Current South African diagnostic and treatment access

In South Africa, access to specialised rare-disease diagnostic services and disease-specific therapies remains uneven across the public and private healthcare sectors [19, 20]. Approximately 84% of the population relies primarily on public healthcare, while 16% has medical-scheme coverage and predominantly accesses private healthcare [21]. South Africa also faces an overlapping burden of communicable and non-communicable diseases, maternal and child health needs, and injuries as the country continues to transition epidemiologically [22, 23]. Decisions regarding high-cost therapies for rare diseases must therefore be considered within a health system managing multiple competing priorities and substantial resource constraints. Limited and geographically uneven specialist medical-genetic capacity may further impede timely diagnosis and referral for rare diseases [24]. These broader inequities have prompted calls for a more coordinated national approach to rare-disease diagnosis and treatment [19]. 

Access to molecular confirmation, lyso-Gb3 testing, cardiac MRI and disease-specific therapy may vary according to geography, referral pathways, healthcare sector and funding arrangements. Based on the authors’ knowledge of the South African service context and consultation with clinicians involved in FD management, access to high-cost therapy remains particularly constrained in the public sector, while private-sector access may depend on individual funding approval and medical-scheme benefit design. ERTs that are not registered in SA may be accessed following SAHPRA authorisation under section 21 of the Medicines and Related Substances Act, 1965 (Act No. 101 of 1965), as amended [25–27]. Some patients may additionally receive treatment through manufacturer-supported humanitarian or compassionate-access programs.

South Africa has no identified coordinated national Fabry registry, broader national rare-disease surveillance system [28] or standardised cross-sector referral pathways. This limits systematic assessment of diagnostic delays, treatment access and patient outcomes. These contextual constraints make a networked model of specialist support, transparent eligibility review and standardised prospective data collection particularly relevant.

## Prioritising disease-specific therapy under resource constraints

In resource-constrained health systems, eligibility for disease-specific therapy cannot be determined solely by the presence of a pathogenic or likely pathogenic GLA variant. Decisions should integrate variant classification, classical versus non-classical phenotype, sex, evidence and rate of Fabry-attributable organ involvement, disease stage, competing cardiovascular and renal risk factors, expected treatment responsiveness, patient preferences and the opportunity costs of long-term therapy [10, 11, 16, 29]. Particular caution is required in individuals with non-classical variants or isolated late-onset cardiac findings, in whom common cardiovascular risk factors or alternative causes of hypertrophy may contribute substantially to observed outcomes [10, 29]. Optimisation of conventional cardiovascular and renal risk management remains essential irrespective of whether Fabry-specific therapy is initiated [30]. 

Evidence for Fabry-specific therapy is heterogeneous and differs by sex, phenotype, organ involvement, disease stage and outcome measured. Available evidence suggests that ERT may slow renal decline in selected patients, with more favourable renal outcomes reported among those with preserved baseline renal function and lower proteinuria, while the magnitude of long-term cardiac and cerebrovascular benefit remains uncertain across several patient groups [11, 15, 16, 31]. These uncertainties should be incorporated into shared decision-making and prioritisation rather than assuming uniform benefit across all genetically confirmed patients.

The reduced responsiveness of established fibrosis and advanced renal or cardiac damage does not establish a universal stage beyond which treatment is futile [12, 14]. Decisions in advanced disease should consider residual modifiable disease, involvement of other organs, anticipated effects on progression, treatment burden, patient goals and competing morbidity. Initiation should be accompanied by explicit treatment goals and a predefined reassessment plan addressing adherence, treatment burden, safety, biochemical response where interpretable, renal and cardiac trajectory and patient-reported outcomes. Treatment discontinuation if poor response is observed should be part of the initiation discussion with patients. Apparent progression does not necessarily demonstrate treatment failure, as it may reflect advanced pretreatment damage, non-Fabry comorbidity or a slower rate of decline than would otherwise have occurred. Continuation, modification or discontinuation requires multidisciplinary review and should not be based on a single biomarker or organ outcome.

High-cost therapy decisions also require transparent consideration of opportunity cost: resources committed to one intervention are unavailable for other health needs. Prioritisation should consider disease severity and unmet need alongside the certainty and magnitude of expected benefit, duration and cost of treatment, feasibility of monitoring, equity implications and budget impact. Definitive national initiation, continuation and discontinuation criteria would require a formal systematic evidence review, health-economic and budget-impact analyses and structured multi-stakeholder consensus; developing such criteria lies beyond the scope of this commentary.

Rather than focusing solely on new context-specific national guidelines, future approaches should prioritise structured implementation mechanisms capable of adapting evolving evidence to resource-constrained environments. These include coordinated national or multicentre clinical networks, strengthened referral pathways, multidisciplinary case review and national advisory or technical oversight to support governance and periodic review. A networked or virtual Centre of Excellence model could be explored as a means of coordinating geographically dispersed expertise, longitudinal follow-up and access to specialised investigations while retaining appropriate local clinical care.

We propose an adaptive, resource-stratified implementation framework developed through formal multidisciplinary and multi-stakeholder consensus. It links three resource levels—minimal, essential and optimal—to explicit referral and escalation criteria, while incorporating expected clinical benefit, treatment uncertainty, equity, affordability and prospective outcome evaluation. The tiers represent resource-linked pathways rather than lower and higher standards of professional care and should be refined as local capacity and evidence evolve.

## Towards an adaptive and resource-stratified implementation framework

### 1. Resource-linked care pathways

Implementation guidance should recognise differences in diagnostic and service capacity across settings. Each tier should specify core assessments, referral triggers, treatment-review processes and feasible monitoring rather than merely listing available technologies.

- Minimal tier: Core clinical assessment and supportive care feasible in highly constrained settings, including symptom and family-history assessment, blood pressure measurement, urinalysis or proteinuria assessment, serum creatinine/eGFR and access to ECG where available. The tier should include clear referral triggers for diagnostic confirmation, specialist review and suspected organ involvement.
- Essential tier: Multidisciplinary specialist assessment; molecular confirmation and expert variant interpretation; structured renal and cardiac evaluation; urine albumin-to-creatinine ratio or proteinuria assessment; eGFR; ECG and echocardiography; disease-specific biomarkers where accessible; and formal assessment of eligibility for, and response to, disease-specific therapy.
- Optimal tier: Comprehensive evidence-informed care incorporating advanced cardiac MRI and other specialist investigations where clinically indicated, access to appropriate disease-specific treatment, pharmacological and supportive care, multidisciplinary follow-up and prospective outcomes evaluation.
- Escalation between tiers: Referral and escalation should be guided by diagnostic uncertainty, disease risk, organ involvement, treatment decisions and changing healthcare capacity. Inability to access an advanced modality should prompt an alternative assessment and referral plan rather than automatic exclusion from care.

### 2. Multi-stakeholder consensus and accountable prioritisation

Framework development should use a formal multi-stakeholder consensus process rather than relying solely on clinician-led recommendations. A national rare disease advisory or technical committee could support consistent evidence review, treatment prioritisation, implementation and periodic reassessment, with representation from:

- Health economists and health technology assessment experts: to assess cost-effectiveness, affordability, budget impact and opportunity cost.
- People living with rare diseases and patient advocates: to incorporate quality of life, treatment burden, travel requirements, preferences and access barriers.
- Policymakers, funders and service managers: to align recommendations with universal health coverage, available capacity and evolving national health-financing arrangements.

### 3. “Living” guideline digital infrastructure

Traditional static guidelines rapidly become outdated. A digital-first living-guideline approach could support responsive and evidence-informed care, provided that responsibility for evidence surveillance, conflict-of-interest management, approval and version control is clearly assigned. Components could include:

- Modular updates: individual sections, including treatment recommendations for females, paediatric patients or particular phenotypes, could be revised as evidence and consensus recommendations evolve without requiring full guideline replacement.
- Clinical decision-support tools: simple digital tools could support non-specialist clinicians with risk assessment, referral pathways and preparation for multidisciplinary treatment review, but should not replace expert interpretation or individual clinical judgement.

### 4. Integration of local real-world evidence

Implementation should be supported by systematic collection and integration of local real-world evidence through low-cost registries and longitudinal data systems, including:

- Outcome audits: clinical outcomes, patient-reported outcomes, treatment exposure and healthcare utilisation should be used to evaluate and refine treatment thresholds, timing, monitoring intensity and continuation decisions across relevant phenotypes and variants.
- Safety and treatment-burden signals: local adverse-event, anti-drug antibody, adherence and treatment-burden data could inform monitoring and shared decisions about ongoing therapy.

**Fig. 1:**
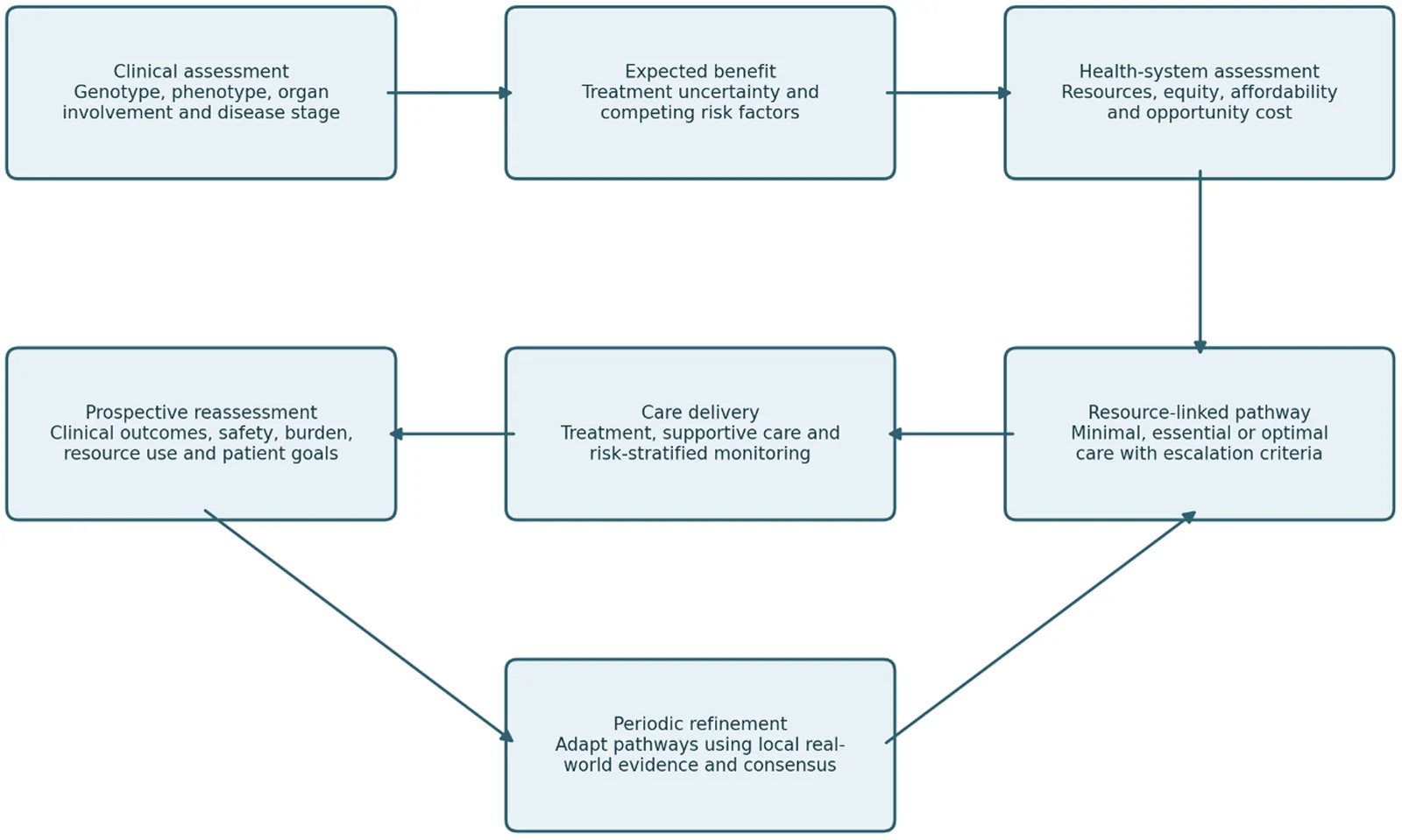
Adaptive, resource-stratified Fabry disease implementation framework

Integral to this approach (summarized in Fig. 1) is standardised prospective data collection capable of capturing phenotype, genotype, longitudinal organ outcomes, treatment exposure and response, patient-reported outcomes, safety, treatment burden and healthcare utilisation. International registries and observational cohorts have contributed substantially to understanding natural history, sex-specific expression and treatment outcomes, but locally generated data are required to assess transferability, equity, effectiveness and resource use in SA and comparable settings [10, 16, 31]. 

These data should feed back into periodic multidisciplinary review of the resource-linked pathways, treatment-prioritisation criteria and referral processes. This adaptive cycle is particularly important where access to specialised diagnostics and therapies varies across settings and evidence is evolving.

## Limitations

This article is a narrative commentary rather than a systematic evidence review, clinical guideline, consensus statement or economic evaluation. The proposed framework identifies principles and processes for implementation but does not establish definitive national treatment eligibility, continuation or discontinuation criteria. Published quantitative evidence on current South African FD services and outcomes is limited; contextual observations should consequently be validated through structured service mapping, stakeholder consultation and prospective data collection.

## Conclusion

Fabry disease management has advanced considerably since publication of the 2015 South African recommendations [6]. Earlier recognition of clinically significant disease, improved assessment of females, genotype-informed care and risk-stratified monitoring have reshaped international practice, while important uncertainty remains regarding long-term treatment benefit across phenotypes, organ systems and disease stages.

The 2015 South African recommendations provided an important pragmatic foundation, but implementation now requires more than periodic replacement of a static guideline. An adaptive framework should link resource-stratified diagnostic and monitoring pathways with transparent treatment prioritisation, multidisciplinary reassessment and prospective local evidence generation. In particular, decisions concerning high-cost disease-specific therapy should consider expected benefit, uncertainty, treatment burden, equity, affordability and opportunity cost.

More broadly, FD illustrates the challenge of implementing evolving rare disease care where specialist capacity and treatment access are uneven. Networked expertise, accountable governance, standardised longitudinal data and periodic refinement may provide a more sustainable and contextually appropriate model than uncritical adoption of international protocols.

Such approaches should address the rare disease equity gap while remaining accountable to wider population health needs, consistent with the 2025 World Health Assembly resolution on rare diseases [5]. 

## Data Availability

No datasets were generated or analysed during the current study.

## Declarations

### Declaration of AI assisted technology

Artificial intelligence (AI)–assisted tools were used to improve the clarity, grammar and readability of the manuscript and to generate Figure 1 based on content and instructions provided by the authors. AI tools were not used to generate original scientific content, analyse data, interpret findings or influence conclusions. Figure 1 and all manuscript content were reviewed and edited by the authors, who take full responsibility for the accuracy, integrity and final content of the work.
